# Conference on Tuberculosis of the Lungs

**Published:** 1919-01

**Authors:** 


VOL. II, No. 6 PARIS JANUARY 1919

### WAR MEDICINE

Published by the American Red Cross Society in France
for the
Medical Officers of the American Expeditionary Forces

### RESEARCH SOCIETY REPORTS

THE NINTH SESSION OF THE RESEARCH SOCIETY OF THE AMERICAN RED CROSS IN FRANCE

November 22 and 23, 1918, at the Hotel Continental, Paris.

### CONFERENCE ON TUBERCULOSIS OF THE LUNGS

MAJOR RIST. We all agree that pulmonary tuberculosis is probably a disease which begins in childhood. What we have to deal with to-day is the question of lung tuberculosis in the adult, and especially in the soldier, — pulmonary tuberculosis from a military point of view.

I think it is evident to all of us that ideas which were current regarding tuberculosis of adults have been to a great extent altered by our experience of war, and I expect this discussion will make a little more clear to all of us the changes which this war experience has brought to us concerning the general evolution and diagnosis of tuberculosis.

1. **What conditions of the naso-pharynx are likely to produce chronic cough, and lead to false suspicions of pulmonary tuberculosis?**

MAJOR RIST. It has been the experience of many of us in the French Army, and I believe also in the American Army, that certain chronic conditions of the naso-pharynx, though often very mild, are able to produce symptoms which have been, and are still mistaken for signs of incipient tuberculosis, and I would like to call on Colonel Emile Sergent of the French Army to say a few words on this point.

COLONEL SERGENT. The war has certainly not changed the clinical history of tuberculosis, but it has brought considerable numbers of documents which have given a much greater experience, and on some points we have come to modify or more clearly define some of our previous opinions on the subject, chiefly on the difficulty in making a differential diagnosis between tuberculosis and a certain number of diseases of a somewhat similar nature.

Among the causes of frequent errors of diagnosis we should bear in mind rhino-pharyngeal affections, especially those obstructing the upper respiratory tract.

All physicians who have examined a great many tubercular patients during the war have noticed a large number of cases among whom tuberculosis was not the disease, but who were suffering with chronic affections of the upper respiratory tract.

Let us examine affections of the nose and pharynx which can lead to this error in diagnosis. It is quite futile to enter into details, and, generally speaking, I think we can sum up the question by saying that all causes of obstruction of the rhino-pharynx are capable of giving rise to conditions which can simulate tuberculosis by keeping up a constant cough and symptoms of bronchitis. This obstruction of the upper respiratory tract has, at first, a mechanical action. The patient whose nose is blocked cannot breathe by the natural way, but must breathe through the mouth. He therefore introduces directly into the bronchial tract dry air, — air which is often cold and has not been filtered through the nasal filter. Apart from the action of cold and dryness which exerts itself mechanically, there is the fact that air is loaded with germs and the latter cause an infection of the mucosa. This infection persists and is, in the case of grippe or cold, warmed up, so to speak, so that this state of chronic tracheo-bronchitis lasts for months and sometimes for years. This is one of the symptoms which simulates tuberculosis to the physician who is not on his guard. We are dealing with subjects which are and will remain " coughers " for many months. If with these subjects one takes the trouble to have recourse to a very simple but safe means of diagnosis, that is to say, examination of sputum, the bacillus in a great many cases is not found, and nine-tenths of them are not tubercular patients.

There is, therefore, a great necessity for knowing such cases. They have been in the course of the war a source of numerous and harmful errors of diagnosis. For my part, I have studied them and I can say that this pathological state, which is extremely frequent in the soldiers at the front, could be classed under the name of " bronchitis of damp feet, obstructed noses ". This is a rather vulgar expression but defines very well a pathological state which is extremely frequent.

That was the point on which I thought it fit to say a few words.

Major Rist. Colonel Sergent has just said that, in his capacity of chief of a triage centre for tuberculous patients, he has seen an enormous number of cases which have been mistaken for tuberculosis and which were nothing but chronic cases of nasal obstruction.

He then went on to analyze why chronic nasal obstruction determines chronic coughing. There is the mechanical side of the obstruction itself, but there is also a certain amount of infection, and there is chronic infection of the upper respiratory tract. It is therefore easy to understand that a great number of people who report for treatment are chronic coughers who have regular attacks of bronchitis.

It is very important to know these facts, and the only way to diagnose them is to have a thorough examination of the expectoration by a specialist. Of course, a man may have chronic nasal obstruction and be tuberculous as well, but repeated examinations nearly always lead to the conclusion that there is no tubercle bacillus in the sputum.

On my own behalf I may add a proof that many cases mistaken for tuberculosis are really cases of chronic nasal obstruction, or of chronic nose diseases which can be given treatment. Before the war and during the war I have seen a great number of cases where the curetting of a suppurating antrum, or any other operation on the nasal organs, has resulted in the stopping of a chronic cough which had lasted for years, and had been mistaken for tuberculosis.

Lieut.-Colonel Webb. Colonel Sergent and Major Rist have had a very wide experience in these conditions, and have called attention to them in their publications. About May, 1917, a letter appeared in the *Journal of the American Medical Association*, signed by Sir William Osler. It called attention to the fact that many hospital beds in France and in England were occupied by soldiers with flat chests, and by many who were mouth-breathers. The letter warned us to avoid sending such men to France, and it guided many of us in our expert examinations of recruits. Some men were discharged, others were referred to specialists in nose and throat conditions, and many were classed for observation.

We noted that one of the most constant causes of chronic cough, especially that of the early morning, was due to the inhalation of cigarette smoke. So much was this the case that we were led to study the effect of cigarette-smoke inhalation on the bronchi as well as on the naso-pharynx. Abundant moist rales, especially those of a bronchial character, could be found in the chests of those who indulged heavily in the cigarette. Physical signs were often present which were mistaken for those of pulmonary tuberculosis. Curiously, such men rarely had, or developed, tuberculosis, and whereas there are probably only about 5 o/o of the men of the American Expeditionary Forces who do not smoke, the following figures are remarkable : — Of the last six hundred men returned from the American Expeditionary Forces to the United States for pulmonary tuberculosis, 32 o/o were absolutely non-smokers, 31 o/o were pipe-smokers, or mild cigarette smokers who did not inhale, and 37 o/o were classed as heavy cigarette smokers. It is possible that in these men, as Sir William Osler remarks in another connection, "there is something wrong with the blastoderm".

At the present time, many cases of chronic cough are being referred to the nose and throat departments for treatment.

Major Rist. I suppose the same experience has been met with in the Italian Army.

Major Paolo Alessandrini : "Yes".

Brig.-General W. S. Thayer. I quite agree with these speakers. I have noted, however, that many such cases have reached the consultant because of associated fever. I have seen a considerable number of patients who showed recurrent fever of a hectic type, suggesting tuberculosis.

The removal of adenoids, and often of infected tonsils, frequently brought about a rapid cure. In private practice I remember an instance of an eight-year-old boy who had nightly chills, with fever to 100°. The removal of the tonsils caused instant improvement, and the child thrived. A year later the symptoms returned. It was then found that a small island of infection had been left, and its removal caused the instant disappearance of fever and a permanent recovery. I feel that the suspicion of pulmonary tuberculosis, because of febrile symptoms, associated with infected tonsils, is a very important incident in the life of the practitioner.

2. **Can conditions resulting from pneumonia and bronchopneumonia, clinically and roentgenologically imitate pulmonary tuberculosis?**

Major Garvin. The right upper lobe possesses one main bronchial stem which soon becomes bifurcated, the upper branch giving off a constant stem — the apicus posterior. The first main branch of the bronchial stem corresponds in surface marking approximately to the first inter-space. It has an anterior and posterior branch. The second branch occupies approximately the surface marking of the second inter-space, and has also an anterior and posterior branch. It also gives off a lower branch to the middle lobe which corresponds approximately to the third inter-space. This also has an anterior and posterior branch. The right lung therefore can be read anatomically as follows : —

| | |
|---|---|
| 1. Bronchus apicus posterior. | 4. Third interspace bronchus. |
| 2. First interspace bronchus. | 5. Interlobar fissure. |
| 3. Second interspace bronchus. | 6. Lower lobe. |

The anatomy of the lower lobe is the development of buddings from the main bronchial stem and presents multiple divisions, but not as definite as the upper lobe.

The left lung presents the same anatomical structure as the right lung, except that the apical bronchus is not anatomically constant and that which corresponds to the third interspace bronchus is not supplied with a constant bronchus. However, the surface markings and general anatomical structure are about the same, but, for the purpose of anatomical accuracy, the areas of lung corresponding to the right bronchus apical posterior cannot be read exactly in this term; also the area of the lung occupying the third interspace cannot be read as having a constant bronchial branch.

The anatomically correct reading for the left lung will therefore be as follows :

| | |
|---|---|
| 1. Left apex cone or area. | 4. 3rd interspace bronchus. |
| 2. 1st interspace bronchus. | 5. Interlobar fissure. |
| 3. 2nd " " | 6. Lower lobe. |

The same markings hold for the left lower lobe as for the right.

*Mediastinum.* Mediastinum should be read from above downward considering the followings points : —

| | |
|---|---|
| 1. Trachea — its position. | 3. Mediastinal curtain — its position. |
| 2. Heart its position. | |

NOTE. The trachea should be noted as central or having a curve to the right or to the left and the exact extent of the deviation.

The heart should be noted similarly if displaced.

The mediastinal curtain should be noted as to the right or to the left in its entire extent or in any section in the upper middle or lower thirds.

Such mediastinal migration will indicate increase in lung volume on one side, and diminution in the other. Such lung volume may be segmentally increased.

*Diaphragm.* The diaphragm should be read as a line across the bottom. Both are equal and active or one is retarded to a definite extent stated in centimeters.

NOTE. In reading, the plate should be read vertically from above downward, the right lung read first as follows : —

*Right lung* : —

1. Bronchus apicus posterior.

2. 1st interspace bronchus.

3. 2nd " "

4. 3rd " ".

5. Interlobar fissure.

6. Lower lobe.

*Left lung* : —
1. Apex area.
2. 1st interspace bronchus.
3. 2nd " "
4. 3rd " area.
5. Interlobar fissure.
6. Lower lobe.

*Mediastinum* : —
1. Trachea.
2. Heart.
3. Mediastinal curtain.

*Diaphragm* : —

Read horizontally.

NOTE. The terms here used indicate the total structure of the lung; the bronchus blood vessel structure which is the unit. By reading the plate in this manner varying degrees of capacity occurring in each of these anatomic divisions may be noted, and also that they may change their position relatively with the surface marking as indicated by the ribs. For instance, the whole upper lobe may be much foreshortened by contraction so that all four of the anatomic divisions of the upper lobe may occupy no greater space than the first two, the upper lobe being much contracted and the lower lobe being accordingly hypertrophied. Similar anatomical manifestations may be observed in segmental parts of the lower lobe.

ILLUSTRATION OF TYPE OF PLATE READING.

*Plate reading. Anterior reading.*

*Anterior or posterior reading* . . . . . . .

*Right lung.*
1. The apical bronchus densely outlined and striated.
2. 1st interspace bronchus, clear.
3. 2nd " " moderately densely striated.
4. 3rd " " clear.
5. Interlobar fissure not photographed.
6. Lower lobe, clear.

*Left lung.*
1. Apex area, clear.
2. 1st interspace bronchus, densely and sharply striated.
3. 2nd " " clear.
4. 3rd " " clear.
5. Interlobar fissure, photographed, normal.
6. Lower lobe, clear.

*Mediastinum.*
1. Trachea bowed to the left one centimeter.
2. Heart, not displaced.
3. Mediastinal curtain, not displaced.

*Diaphragms* : Equal and active.

*Posterior reading.*

The same rules can be observed for reading a posterior plate as for an anterior plate. The same anatomical divisions can be traced from the corresponding interspace as shown at the periphery of the plate to the root of the lung.

*Standardization of position for photography.*

1. Distance of tube from plate 28 inches or more, but exact distance should be determined and made standard for all work.

For position of viscera only, without fine details, distance 6 feet.

2. The tube should be centered in the exact middle line; this will obviate distortion caused by taking oblique photograph.

3. The tube should be centered vertically at the manubriogladiolar junction for posterior photograph and at the fourth or fifth spinous process for anterior photograph.

4. Position of the patient: Lying or standing. It would seem best for uniform work to standardize the position as either standing or lying. As probably most of the work will be done lying it might simplify matters to have it all done lying for both anterior and posterior positions. Head always in the median position, and not turned to right or left.

5. All photographs should be taken on held breath if possible.

SCHEME FOR PLATE READING

| Right. | Mediastinum. | Left. |
|---|---|---|
| 1. Post apical bronchus.<br>2. 1st interspace bronchus.<br>3. 2nd interspace bronchus.<br>4. 3rd interspace bronchus. | Position of Trachea.<br>Heart.<br>Mediastinum. | 1. Apical area.<br>2. 1st interspace bronchus.<br>3. 2nd interspace bronchus.<br>4. 3rd interspace area. |
| Interlobar fissure. | | Interlobar fissure. |
| Lower lobe. | | Lower lobe. |
| Diaphragm. | | Diaphragm. |

MAJOR RIST. We are very thankful to Major Garvin for his interesting scheme of standardization for the reading of X-ray plates. I believe an interesting subject for future conferences on tuberculosis would be one in which the standardization of the reading of these plates could be taken up. I will now call upon Captain V. C. Vaughan, who will show us some interesting plates of pneumonia and broncho-pneumonia cases, which have simulated pulmonary tuberculosis.

CAPTAIN V. C. VAUGHAN. — The question is whether or not one can have conditions arising from pneumonia and broncho-pneumonia which cannot be distinguished from tuberculosis. Primarily one would think it impossible. However we have had such conditions at Base Hospital No 15, which I shall illustrate to you on the screen.

The first plates are of lobar pneumonia. Major Steiner and I have found that the right upper lobe is very frequently involved, often first, in lobar pneumonias.

Recently we have had a case of acute tuberculous pneumonia in the right upper lobe, which, as you may see by the lantern slides, puzzled us exceedingly until tubercle bacilli were found in the sputum.

MAJOR. RICHARD C. CABOT. In the 21738 patients received in Base Hospital 6 between September 1917 and November 22nd, 1918, there were 63 positive cases of tuberculosis, pulmonary or extra-pulmonary. Of these, 51 were recognized by the presence of tubercle bacilli in the sputa and 12 were found postmortem.

There were in addition 100 cases diagnosed as probably or possibly pulmonary tuberculosis, no other diagnosis seeming more likely — bacilli not found in the sputa.

All these 163 cases were apparently of advanced, none of incipient tuberculosis.

The incidence of tuberculosis was greatest in the earliest months, when presumably the "combing-out" of tuberculous cases by special examination in the training camps of the United States had not begun, or was not extended to all units. Stevedores, labor companies and engineers were especially affected, and I am told that in many of these units the men received no careful medical examination. The distribution of tuberculosis in each 1000 patients received by this hospital is shown in the following table :

| Patients received. | Cases of positive pulmonary tuberculosis. | Patients received. | Cases of positive pulmonary tuberculosis. |
|---|---|---|---|
| First 1000 | 2 | 12th 1000 | 4 |
| 2nd " | 7 | 13th " | 3 |
| 3rd " | 4 | 14th " | 2 |
| 4th " | 10 | 15th " | 1 |
| 5th " | 8 | 16th " | 3 |
| 6th " | 2 | 17th " | 1 |
| 7th " | 2 | 18th " | 0 |
| 8th " | 0 | 19th " | 0 |
| 9th " | 0 | 20th " | 0 |
| 10th " | 0 | 21st " | 0 |
| 11th " | 2 | 22nd " | 0 |

Thus it appears that in the first 7000 cases treated in Base Hospital No. 6, there were found 35 out of the 51 positive tuberculous cases, while in the last 6000 cases received only 1 case was proven tuberculous.

Few, if any, cases could have been fairly considered as originating in line of duty. The great majority of cases probably existed prior to enlistment. Other infectious diseases, such as measles, pneumonia, broncho-pneumonia and influenza seemed to play little or no part as pre-disposing causes in the development of phtisis among our hospital patients. Gas poisoning at the front was also without causative influence so far as we could ascertain.

CONCLUSION. 1. Pulmonary Tuberculosis was of rare occurrence among the sick treated at Base Hospital No. 6.

2. It occurred chiefly among soldiers who had not been specially examined in the training camps of the United States with reference to its presence.

3. Even three fifths of the 51 cases with tubercle bacilli in the sputa occurred in the cases between No. 1 and No. 7000 of our series. Only 1 such case was found in the cases numbered from 17,000 to 21,723.

4. The predisposing influence of infections or gas irritation was not discernible in our series.

5. No incipient cases were recognized.

LIEUT.-COLONEL WEBB. Three cases which were returned from the American Expeditionary Forces as pulmonary tuberculosis are now being returned a second time to the United States. It is very natural that the same difficulties in diagnosis should have occurred in the American Expeditionary Forces as occurred in our camp examinations in the United States, when many cases of delayed convalescence from pneumonia and broncho-pneumonia were mistaken for tuberculosis. In one case which was considered pulmonary tuberculosis and sent back to the United States, the diagnosis could not be confirmed there. This man was again sent to the front, and recently died of peritoneal tuberculosis. The autopsy revealed no tuberculous lesions of the lungs, but the intestines, mesentery and diaphragm were studded with miliary tubercles.

There have been many of our soldiers in France who have had what might be termed abortive pneumonias and broncho-pneumonias. Usually such were not admitted to hospitals and often only presented symptoms of chest-colds for a day or two. Such men had occasional bouts with fever and a residual fraction of their infections could be detected in their chests. In examining men who have had definite pneumonias, I have been frequently struck with the retraction of an apex and the lagging motion of that side of the chest. Inspection of such chests immediately suggested pulmonary tuberculosis, but careful observation of the temperature and pulse, the repeated negative sputum examinations, and the gradual disappearance of indeterminate rales usually cleared up the diagnosis.

Pathologists, such as MacCallum, have called attention in their studies of autopsies to the close resemblance to tubercle of patches of broncho-pneumonia of the interstitial type.

The beaded appearance seen in the X-ray plates has closely simulated tuberculosis, and it is only by taking a series of plates at varying intervals that the differential diagnosis of pulmonary tuberculosis from the broncho-pneumonias has been established.

Major C. Bjerving. The plate I wish to show you on the screen is that of a soldier who was admitted to Base Hospital No. 20 for observation for tuberculosis. The man was about 32 years old and gave a history of a definite hemorrhage six years ago. The physical findings were those of a healed, fibroid tuberculosis at the right apex. The routine X-ray examination reveals, as you see, a series of small, definite cavities throughout the upper right lobe. The arrangement of these is somewhat similar to a honeycomb. Should it be a case of tuberculosis, it is certainly an unusual finding.

Captain J. H. Musser. This soldier is still under observation, although he appears to be in perfect health. There have been occasional slight amounts of sputum which have been investigated for every condition. The sputum has been repeatedly negative to tubercle, to streptothrix, to moulds, to hydatid material. The Wasserman test is negative.

Captain W. A. Somerville. We have received at Savenay, which is the final evacuation centre for cases of tuberculosis, many slowly convalescing pneumonias. These men show generally a loss of weight, a low grade of temperature, and cough which varies from day to day. The X-ray plates always show a marked thickening of the bronchial tree, which is especially confusing in the pneumonias which have involved the upper lobes. Postural drainage has been most valuable in these cases which have shown a tendency to bronchiectasis. These men finally conquer their bouts with fever and are then evacuated to the Convalescent Camp where, under Captain Morrison, they are very gradually and carefully restored to their commands. Repeated negative examinations of sputum, especially when considering the extent and type of the rales auscultated, should always suggest that in spite of the corroborative X-ray findings, these cases are not tuberculous.

Major Rist. I quite agree that the difficulty in distinguishing these forms of broncho-pneumonia from tuberculous broncho-pneumonia is not only met with clinically and roentgenologically, but it is also met with on the post-mortem table. I have seen specimens of these types of broncho-pneumonia following measles in the United States Army, which at first sight could not absolutely be distinguished from tuberculosis, and it was only on close bacteriological examination that they showed signs of the streptococcus hemolyticus. We may now close the discussion on question 2. The frequency of errors of diagnosis due to naso-pharyngeal conditions and pneumonias are lessons of the War.

3. **What heart conditions are apt to be found in the tuberculosis observation centers?**

Lieutenant-Colonel Alfred E. Cohn. Two conditions involving the diagnosis between tuberculosis and heart conditions may arise in Tuberculosis Observation Centres. The first involves chronic heart disease and especially mitral stenosis. Few cases of this disease came under observation because the cases were recognized and discharged from the army in the United States. Scarcely 50 cases of organic heart disease have come under my personal observation in the A. E. F. The importance of mitral stenosis in this connection rests on the fact that the first sign, as in tuberculosis, is often haemoptysis. The distinction between the two diseases need present no serious difficulty when a satisfactory physical examination is made. The other affection which might cause confusion is the effort syndrome. This is a neurosis of frequent occurrence in armies. It is characterized usually aside from the nervous manifestations by rapid heart rate, a sense of fatigue or weakness, shortness of breath and tremor. Here again the history and the physical examination should clear the difficulty in diagnosis. It is of course possible that tuberculosis and the effort syndrome may coexist.

Captain W. A. Somerville. Cases of effort syndrome have not been uncommon at our center. They have been passed from hospital to hospital with the diagnosis of tuberculosis. Careful observation has decided the diagnosis, and many have been returned through the convalescent camp to the front. Only one or two cases of mitral stenosis and of aneurism have been seen. Apnoea is a symptom curiously characteristic of both effort syndrome and tuberculosis.

4. **Do we expect to find pulmonary tuberculosis following gas or thoracic wounds?**

MAJOR RIST. In many cases of thoracic wounds chronic bronchitis has been found, as well as chronic lung infections, and these have naturally raised a question of tuberculosis. Bronchial pneumonic conditions following gas have been mistaken for tuberculosis and only after a long time have the errors been recognized. The experience of the French Army is that tuberculosis very seldom follows thoracic wounds and gas poisoning.

MAJOR FLANDIN. I am bringing you not only my own experience but also that of Professor Achard. In the French Army we have had to deal with asphyxiating gas since April 1915, and with mustard gas since August 1916. I quite agree that gas does not produce chronic tuberculosis, but there are cases where old tuberculosis recurs in the first acute stage of gas poisoning, and in about a fortnight becomes active. There is difference between the sequels of suffocating gas poisoning and the sequels of ether poisoning.

The patients sometimes seem to recover, but on returning from convalescent leave it is noticed that they are absolutely unfit for duty. They have fever, effort syndrome, at night-time they have fits of coughing, and they get thinner and thinner and have to go back to hospital. This condition may last for months, and on examination we find that these men show signs of bronchitis, of bronchial dilatation, or lobar congestion at the apex or base. One special feature of etheric poisoning is that there are signs of hypertrophy of the thyroid gland, and by examination one finds signs of disseminated shadows which remind one of dilatation and sclerosis of the bronchi. Examination of the sputum, which is very abundant after coughing, especially in the morning, shows no signs of tuberculosis — you never find the Koch bacillus in the sputum. Inoculation to guinea-pigs is negative as a rule, and the evolution of the case is quite against the idea of tuberculosis, because after a period of 7 to 18 months, or even two years, most cases simply recover, the fever falls very slowly, the patient's weight increases and he recovers, but not so completely as to be fit for heavy muscular work; sufficiently however for agricultural work or easy work in a factory.

Experimentally, we know that individuals poisoned by gas are not more susceptible to tuberculosis. We poisoned some guineapigs in this way and placed them with as many tuberculous guinea-pigs. After several months there was exactly the same number of tuberculous guinea-pigs in both lots. By poisoning dogs we obtain the same symptoms as those shown by men; the dog loses weight, gets tired after a short run, has fits of coughing, and the $CO_2$ test gives a very characteristic curve, in the acute stage. When the animal recovers, the $CO_2$ rises again and sometimes becomes higher than the normal level. The period for recovery varies between 3 and 12 months. This shows that after gas poisoning one must get accustomed to the new condition of the lungs. Besides the symptoms which are noticed at once, in some cases there is emphysema, etc. Histological examination shows a decreasing of the elastic fibers and an increasing of the connective tissue.

The general conclusion is, therefore, that gas poisoning does not predispose to tuberculosis. With men who are convalescent after gas poisoning, we have to consider a kind of training of the lung to get it accustomed to its new condition. This brings up the question of the best exercise for gassed patients and that of oxygen treatment, but this is a little out of the scope of the discussion.

Major Rist. Professor Alessandrini, of the Italian Army, asks me to say that out of 10,000 cases under suspicion of tuberculosis he has not seen one single case which he could positively assign to gas poisoning.

Major Léon Bernard. I have studied the question at the casualty clearing service of Laennec hospital. The results of my work have already been published by the Société des Hopitaux and I shall but summarize them here. My studies were based on the observation of 475 cases of pulmonary traumatisms, including 379 chest wounds, 47 gas cases and 49 cases of thoracic contusions. In the 379 chest wounds, 7 bacillary collections only were found : 5 closed and 2 open. When we endeavored to determine the connection between tuberculosis and chest wounds, the following questions arose :

1) What proportion of chest wounds develop tuberculosis?

2) What is the part played by pulmonary tuberculosis in chest wounds?

In order to solve the first problem, we have studied the question on outdoor patients : of 379 cases with chest wounds only three were complicated by pulmonary tuberculosis, which gives a proportion of 1 per cent.

In order to answer the second question, we have, for the certainty of the diagnosis, included in our statistics only the tuberculous patients showing bacilli in the sputum. The proportion of old chest wound cases with tuberculosis was 2.1 per cent.

Of 44 gas cases observed in 1916, only one was suffering from tuberculosis, while proportion of tuberculous patients in cases of thoracic contusions was found to be 6 per cent.

The following conclusions were therefore drawn.

1) The influence of chest wounds on the development of tuberculosis is very small.

2) The inhalation of toxic gas plays no important part in the history of tuberculosis.

3) Thoracic contusions are more liable to give rise to tuberculosis. However these 3 causes have but an insignificant role in war tuberculosis.

The above conclusions have been unanimously accepted in the French army.

Lieutenant-Colonel W. Malloch Hart. In some eight or nine hundred cases of pulmonary tuberculosis which have come under my observation in the Canadian Army, there has been only one case of wound of the chest, and this wound had nothing to do with the tuberculosis. In the comparatively few cases where patients suffering from tuberculosis had been exposed to gas poisoning, in no single case could we conclude that this had been a factor in producing the disease.

Lieutenant R. J. Allison. I have stereoscopic plates to show later to the meeting of cases of broncho-pneumonia mistaken for pulmonary tuberculosis, and also a case of miliary tuberculosis of the lungs following a shrapnel injury to the left shoulder and chest. Colonel Webb tells me this is the only one of the kind he has seen in the American Expeditionary Forces. The soldier, aged 22, had never had symptoms indicative of pulmonary tuberculosis. The injury was immediately followed by haemoptysis, and the chances are that the miliary condition was hematogenous.

Major A. P. Francine. I have been in charge of an extensive gas service. I have detected no case of pulmonary tuberculosis resulting from gas. In cases which have come to autopsy there has been no evidence of a tuberculous process being awakened by gas.

Lieutenant-Colonel Webb. In 700 cases which have passed through Savenay, of which 84 o/o have had sputum positive to tubercle, there have been only twenty cases with a history of being gassed, and in these we had to take the men's word concerning their exposure. Most of these cases had coughed previously to the gassing. It would seem, therefore, a very rare occurrence for pulmonary tuberculosis to follow gas poisoning.

Major Rist. We have other "wounds" of the chest besides those referred to, and I will ask Captain Somerville to tell us the incidence of tuberculosis following pneumonia and influenza.

Captain W. A. Somerville. In 14 o/o of our cases, there has been history of pneumonia. Such histories from soldiers may signify that exacerbations of tuberculosis have been present and not necessarily genuine pneumonia.

Influenza broke out with us in the late spring and again more seriously in late September. A proportionate increase in the number of tuberculosis cases followed each of these outbreaks, as will be noted in the following figures, due allowance being made for the great increase in the numbers of troops : — 84 o/o of the following numbers of cases had sputum positive to tubercle.

*Men with Pulmonary Tuberculosis Returned to the United States.*

| | |
|---|---|
| April | 47 |
| May | 59 |
| June | 112 |
| July | 162 |
| August | 137 |
| September | 102 |
| October | 247 |

For November, as far as at present can be estimated, we shall have considerably less than 200.

5. **What other diseases are mistaken for pulmonary tuberculosis?**

Captain W. A. Somerville. In the order mentioned, the following conditions have come to us with the diagnosis of tuberculosis observation.

1. Gastric duodenal conditions with the loss of weight, cough, asthenia, and shortness of breath.
2. Hyperthyroidism.
3. Psychoneuroses.
4. Syp hilis of the Lung and New Growth.
5. Bronchitis, due to the Klebs-Loeffler bacillus.

The latter cases all cleared up under the use of antitoxin. We have found no case of streptothrix.

Major Rist. I can quite agree with Captain Somerville that cases of hyperthyroidism are very apt to be mistaken for pulmonary tuberculosis. These cases may present an irritative cough, emaciation, shortness of breath, and fever.

Lieutenant-Colonel Aldo Castellani. I might perhaps say a few words on two affections which are very often mistaken for pulmonary tuberculosis, viz., broncho-spirochaetosis and bronchomycosis.

Broncho-spirochaetosis is a disease described in Ceylon in 1905 and 1906, my work being later confirmed in practically every tropical country. The condition, however, attracted very little attention in Europe until the recent important researches of Galli-Valerio in Switzerland and Violle in France. The first case in Europe was placed on record by Lurie in 1916. The affection is due to a very polymorphitic spirochaete which I found in 1905, and named S. bronchialis in 1907. The organism has been recently investigated very completely by Professor Fantham, who has conclusively demonstrated that it differs from all the usual mouth spirochaetes.

Professor Fantham has discovered the coccoid stage of the organism.

*Clinically*, three types of disease may be distinguished :

1. The acute stage.
2. The sub-acute stage.
3. The chronic stage.

In the *acute type* the patient feels chilly and develops fever which generally lasts between three and six days. He complains of rheumatoid pains all over the body, and coughs a great deal, the expectoration being scanty, mucous or muco-purulent, This type somewhat resembles influenza.

It is the *sub-acute and chronic types* which may closely simulate pulmonary tuberculosis, as in these types the patient frequently spits up *blood*. In certain cases there may be serotine fever, and the patient may waste. On the other hand, in a fairly large number of cases, the general condition remains very good, and is greatly in contrast with the presence of broncho-pulmonary hemorrhages.

The diagnosis is based on the presence in the sputum of very numerous spirochaetes of the bronchialis type, while the search for T. B. remains constantly negative. A precaution which must always be taken is to make the patient rinse his mouth and gargle with sterile water, before telling him to cough and bring up some phlegm. This is done with the object of getting rid of the mouth spirochaetes as far as possible. Fresh preparations can be examined with the ultramicroscope; films may be stained with one of the many modifications of Rornamowsky's method, or a nitrate of silver staining method, such as the Fontana-Treboudeau, may be used.

The prognosis is, on the whole, favorable quoad vitam, but recurrences are extremely common, and the condition may last for years with periods of apparent cure. The first case I saw in Ceylon in 1905 was still alive when I left the island in 1915.

As regards treatment, a change of air with rest and plenty of good nourishing food is all that is wanted in many cases. In certain ones, especially those of the sub-acute and chronic type, arsenic will be found very useful, and at times a mixed arsenic tartar emetic treatment is of great advantage.

The term broncho-mycosis covers a number of etiologically different conditions which, however, are clinically very similar. They may be classified as follows:

1. Due to fungi of the genus Nocardia.
2. Due to fungi of the genus Monilia and of the genus Oidium.
3. Due to fungi of the genus Hemispora.
4. Due to fungi of the genus Aspergillus, genus Sterigmatocytostis, genus Penicillium, genus Mucor.
5. Due to fungi of the genus Sporotricum and genus Acladium.
6. Due to fungi which have not yet been definitely classified.

Whatever the fungus presents, the clinical symptoms are very similar, and from a practical point of view two types of the condition may be separated.

1. The mild type.
2. The severe type.

In the mild type, the patient has the usual symptoms of a mild attack of bronchitis which generally gets cured in a few weeks.

The severe type may closely resemble tuberculosis. The general condition of the patient is very poor, he feels weak, has serotine fever and may waste rapidly. There is cough with mucopurulent expectoration which may be tinged with blood. At the physical examination of the chest, patches of dulness may be found in certain cases, and crepitations detected. The course is variable, and the malady may end in death.

The diagnosis is based on the microscopical and cultural investigation of the sputum collected with all due precautions.

The prognosis depends a great deal on the species of fungus found, and on the general condition of the patient. If the condition is of nocardial origin, the prognosis is bad; if of monilia or oidium origin, the prognosis is less unfavorable; if of hemispora or of sporotrichum origin, or in general due to fungi rapidly influenced by potassium iodide, the prognosis is good.

The treatment consists in giving the patient large doses of potassium iodide, which, however, as I have already stated, acts only on certain fungi, not on all.

In conclusion it seems to me that more attention should be paid to broncho-spirochaetosis and broncho-mycosis than has been done so far, the subject having a certain importance, not only from a purely scientific point of view, but also from a practical point of view.

The reader who desires to have more information on the subject of broncho-spirochaetosis and broncho-mycosis might perhaps find it of advantage to consult my publication in the *Presse Médicale*, No. 37, 1917, and *Journal of Tropical Medicine*, August and September numbers, 1917, as well as Galli-Valerio's paper in *Correspondenzblatt fur Schweizer Aertze*, February, 1917, and Violle's recent paper in the *Presse Médicale*, August, 1917.

Lieut.-Colonel Webb. Glover, of the Birmingham Sanatorium, by a most thorough and careful analysis of the cases sent to his institution, has shown that 62 o/o are not tuberculous.

This, I believe, would agree with the findings of most sanatoria men, and it is therefore not to be expected that Army medical officers would be able to do better. This is about the percentage of error that is found at the three Centres de Triage.

6. **To what extent is the tubercle bacillus the etiological factor in the pleural effusions seen in the American Expeditionary Forces ?.**

Major Cabot. It has appeared quite striking that so many non-tuberculous pleurisies have hung on in a way to make us suspect tuberculosis but which careful investigation proved non-tuberculous. Most of us, I am sure, would have said that most of the pleurisies we would see were tuberculous, but it would seem we have now learned something more about differential diagnosis.

Major Rist. I have felt that all the pleural effusions I have seen at the front were due to the tubercle bacillus and I very much question whether it is possible to state that many pleural effusions are not tuberculous.

Lieut.-Colonel Webb. We have had a large number of pleural effusions, and for various reasons I have gained the impression that the majority of them have not been due to the tubercle bacillus.

Last spring we returned about fifty to duty, and, up to the present, not one of these men has returned to a tuberculosis center. It has been exceptionally rare to find a tuberculous deposit in the lung opposite to that affected. In some, the streptococcus has been found in the aspirated fluid. Owing to a lack of mice and guinea-pigs we have not been able to make the investigations we had planned. Nine percent. of the seven hundred cases returned to the United States have, it is true, given a history of pleurisy with effusion. An attendant in a pneumonia ward developed pleurisy with effusion, which proved not to be tuberculous. Major R. C. Cabot and Major F. A. McGraw have made an attempt to study this question and are under the same impression, that the tubercle bacillus is not the chief etiological factor in these pleural effusions.

7. **In the positive diagnosis of pulmonary tuberculosis what weight should be attached to** :

a) *History?* b) *Sputum examination?* c) *Physical and clinical examination?* d) *X-ray findings?*

LIEUT.-COLONEL WEBB. In the camp examinations and in the examinations at Savenay, there have been only two points worthy of consideration. 1. Did the soldier's father, mother, sisters or brothers have pulmonary tuberculosis, and if so, did he live with them? Thirty-six percent. replied in the affirmative. 2. Did the soldier ever spit as much as amouthful of blood? Fifty-six percent. replied in the affirmative. Slight hemorrhages amounting to about half an ounce are characteristically early signs of pulmonary tuberculosis as recorded in four thousand cases at the Brompton Hospital.

One must always be certain that the sputum comes from the soldier in question. Men have been known to put the sputum of tuberculous soldiers in their mouths so that they might get a discharge from the Army. Captain Somerville detected one soldier who was swapping sputum by the simple expedient of placing a little phenolphtalein in the cup. The addition of an alkali later told the story.

In suspicious cases concentration methods should always be employed. The anti-formin method or the concentration method of Ellerman and Erlandsen are the best. The latter has been found to reveal tubercle bacilli in forty percent. of sputa otherwise negative. Specimens should always be examined by the Gram method as well as by the Ziehl-Nielssen. In this way the influenza bacillus, the Friedlander bacillus, and the Klebs-Lœffler bacillus, will be the easier detected. All these bacteria can cause lung conditions simulating tuberculosis.

The study of the temperature and the pulse are of the greatest importance in all types.

The rapid examination of the soldier with auscultatory cough has proven to be a method of great value in detecting probable lesions of the lungs. By this means, seventy-five to one hundred men can be examined daily by an expert. It is a method available to overworked ward surgeons. The man is instructed to breathe moderately in, out, and with the last outgoing breath to give a short sharp cough. Careful auscultation during this procedure reveals changes in the breath sounds, crepitant and sub-crepitant rales, and indeterminate typical and atypical rales, all characteristic of the different types of tuberculosis. The method as advocated by Colonel Bushnell is to begin the examinations at the bases, standardizing the vesicular breathing in the axillae, and proceeding upward to the apices. Inasmuch as the lesions of tuberculosis are usually in the upper lobes, one proceeds by this method from physiological conditions to pathological. Lesions detected in this rapid manner can be studied more thoroughly later. In Circular No. 20. S. G. O., will be seen the details and the suggestions relative to this method. The detection of rales, especially if limited to definite regions, suggests a very thorough search for tuberculosis.

There are many other pathological lung conditions, in addition to those seen to-day, which may imitate quite closely many X-ray findings in the tuberculous. There is probably only one very definite feature characteristic of tuberculosis alone in an X-ray plate, namely, thickened and beaded peribronchial thickenings, extending to the periphery and there ending in a "golden-rod burst". Circular No. 20 S. G. O., should be referred to by those interpreting X-ray findings in the tuberculous. One has seen sarcomatosis and broncho-pneumonia mistaken for miliary tuberculosis.

8. **What type of pulmonary tuberculosis may we expect to see in the army?**

The types seen have been :

1. Parenchymatous or alveolar type.
2. Peri-bronchial or para-vertebral type.
3. Miliary type.

Probably nearly 60 cases of the miliary type have been recognized and confirmed at autopsy. X-ray evidence in one soldier demonstrated a complete recovery from a slight ancient miliary condition. One distressing case occurred in a young lieutenant immediately following the removal of his tonsils, and death occurred within five weeks.

In a recent publication by Colonel Bushnell an analysis was presented of 984 tuberculosis patients studied by the roentgenogram.

394 had definite para-vertebral or peri-bronchial or the lymphatic type.

2 cases alone showed tubercles present at the apex without peri-bronchial shadows.

82 showed para-vertebral tuberculosis alone as revealed by thickened lines with studding extending towards or to the apices.

In 39 of these eighty-two the lines had not reached the apex.

45 of these eighty-two cases gave no definite physical signs.

All 82 soldiers showed at some time symptoms such as cough, expectoration, pleurisy or hemorrhage, and 80 had at some time tubercle bacilli in their sputum.

Of 62 incipient cases 37 had no physical signs, yet all had, or had had, symptoms, and all except one had, or had had, positive sputum.

All 62 cases, except 2, showed para-vertebral tuberculosis above the hilus, and it was considered, therefore, that one was dealing with conclusive evidence of tuberculosis of the deep lung in its early phases. Colonel Bushnell concludes that incipient tuberculosis is not incipient, but is detected when the deep peri-bronchial tuberculosis invades the superficies, or, in other words, when tuberculous lymphangitis or peri-bronchial pneumonia extends into a larger tuberculous broncho-pneumonia involving the cortex of the lung, the so-called primary tuberculosis.

Heise and Sampson also called attention to these different types of pulmonary tuberculosis. They term the peri-bronchial or para-vertebral the closed lymphatic type, and state that in these cases hemorrhage is less frequent, tubercle bacilli in the sputum are less frequent, the complement fixation test is less apt to be positive and the Von Pierquet reaction is apt to be less positive.

Colonel Bushnell, who for sixteen years at Fort Byard has so carefully followed all post-mortem examinations, states that the peri-bronchial thickenings due to tubercles are very easily overlooked by pathologists.

9. **In what lobes should we look for tuberculosis lesions, and why?**

LIEUT.-COLONEL WEBB. As Theobald Smith has so often insisted, we should look at the question of pulmonary tuberculosis more from the parasite point of view. In order to perpetuate their species, parasites must have access to and egress from their host.

We may, in the language of the day, look at the tubercle bacillus as desiring " a place in the sun ", domination and not destruction of " colonies " where it can continue its " militarism ". As one enemy of his type has so recently been vanquished, let us now concentrate on the destruction of this other terrible foe, the tubercle bacillus. As Theobald Smith explains, the tubercle bacillus is " tuned-up " to the human adult lung, all other forms are aberrant. It is not yet decided how the tubercle bacillus reaches the lungs, whether by the inhalation route (favored by French authorities), by the intestinal route (favored by German authorities), or by the nose and throat, especially through the tonsils, a route which may in time be considered more important. Possibly all routes lead to the hilus where we find so often the tubercle bacillus established. To achieve its "national aspirations" it has probably on account of anatomical and physiological chest conditions established definite routes of egress. It is of especial interest that surgeons like Robinson have in recent years traced tuberculosis of the ribs and sternum to pleural and mediastinal tuberculous deposits. We have seen cases where the tubercle bacillus has succeeded in escaping from the chest by the surgical as well as the medical routes.

Opie has attempted to show that the apical infection of adults is a new infection probably from without, and not necessarily connected with the focal infection of childhood. Opie demonstrated that the lymphnodes connected with the apical infection were usually not caseated, whereas those connected with the focal infection of childhood were usually caseated. Opie compares these findings with the well-known results of Koch in the inoculations of guinea-pigs. Opie fails to explain, however, why tuberculous lesions of adults are chiefly in the upper lobe. Of 700 definitely tuberculous soldiers, the disease was present in the right upper lobe in 140 men, and in the left upper lobe in 80; in all the others, the disease was disseminated. In regard to the focal infection of childhood, Ghon found the deposits in the proportion of 5 for the lower lobes to 7 for the upper. He pointed out that the inoculation points in the lung might entirely disappear, leaving only the hilus lymphnode infection. In 17 o/o of tuberculous children, Ghon found at autopsy a bronchial node breaking or broken into a bronchus. Harbitz found a similar condition in 20 o/o. Tenderloo has most thoroughly studied the mechanical forces which operate the chest. In brief, it is pointed out that above the bifurcation of the trachea, there is very little movement of the chest, and that the lymph flow in the upper lobes is very sluggish. On account of the pumping action of the diaphrágm, the lymph flow in the lower lobes, especially in the peribronchial lymph-spaces which are perpendicular to the diaphragm, is very swift. The para-vertebral region from the fifth rib upwards would also seem to be a region of biochemic susceptibility to tubercles.

Should we accept the ruptured lymphnode theory as suggested by Ghon's work, the purulent material scattered by cough throughout the lungs would be rapidly swept away from the lower lobes, the cleaning process there being so great, whereas the material forced to the apex would stand a very much better chance of developing infection. The more probable theory of the tubercle bacillus, travelling from the hilus by the peri-bronchial lymph-spaces to the upper lobes on account of the more sluggish lymph movement, fits in best with the studies of Colonel Bushnell as reported above. There can be no doubt that the apical infection of adults increases with age, although active tuberculosis does not necessarily. After seventy years, fifty percent. of autopsies revealed definitely tuberculous upper lobes.

10, **What can we learn regarding pulmonary tuberculosis from the opportunity afforded by the general postmortem ?**

Major Henry W. Cattell, M. C., showed lantern slides giving data concerning the prevalence of tuberculosis in the American Expeditionary Forces, as determined from the autopsy protocols received by the Director of Laboratories. He called especial attention to the danger of drawing wrong conclusions from these statistics, as the mortality rate of tuberculosis was naturally lowered when the total death rate was increased by those dying from wounds or from the present widespread epidemic of influenzal pneumonia. An analysis of the autopsy protocols recently received indicates that tuberculosis is the direct cause of death in a little over two percent. of those soldiers who come to autopsy. It is to be expected that the percentage of deaths from tuberculosis will increase in the near future, both from the cessation of hostilities and from the escape of tubercle bacilli from foci harmless up to the time of an influenzal pneumonia from which the patient will make an apparent recovery.

Major D. J. Glomsett. Out of 308 autopsies I have performed at Base hospital No. 15 only 6 deaths resulted from tuberculosis. I found about the same proportion in the autopsies at a cantonment in the United States. I am struck with the rarity of deaths from pulmonary tuberculosis. It is extremely interesting that such a small percentage of our soldiers show any evidence of tuberculosis at all. In my experience only 44 have shown any lesion whatever. Tuberculous lesions are certainly rarer in young men than they are in men over fifty. Probably eleven percent. of our autopsies have shown definite deposits of tubercle, whereas in men of over fifty, one would certainly find 60 percent. with deposits. Such evidence hardly supports the view that all infection is in childhood, and I feel it may be as easy to become infected with tubercle in later life as with other diseases. The vast majority of my cases have shown what I call a primary focus in the lung, with a secondary deposit in the hilus. To my mind, tuberculosis travels from the lung to the mediastinum. I have only seen one case in which I did not succeed in finding a primary focus in the lung. I have found the lesions in the upper lobes and in the lower lobes, especially on the left side, as well as in other parts of the lung.

Major H. E. Robertson (Communicated). During the first year of the war, I worked in Germany at Freiburg with Aschoff, and I was struck with the high percentage of lesions of tuberculosis found in the German soldiers. In 100 autopsies which I made for Pick, 70 o/o revealed the deposits in the lungs or nodes, especially in the nodes at the bifurcation of the trachea. It has seemed quite remarkable to me that in making autopsies on our soldiers, I have been unable to detect deposits of tubercle in even as much as 25 o/o of the cases.

Captain A. N. Desjardins (Communicated). Out of a series of 82 deaths from all causes, I have found gross evidence of tuberculosis in 20, as shown in the following chart. A point worthy of note is that, with one or two exceptions, the lesions were in the left lung and usually in the lower lobe.

| | Acute. | Fibrous. | Chronic Calcareous. | Miliary. |
|---|---|---|---|---|
| Lungs. . . . . . . . . . . | 0 | 1 | 11 | 0 |
| Trach.-bronch. Lymph. n. . | 0 | 0 | 7 | 0 |
| Liver . . . . . . . . . . | 0 | 0 | 0 | 4 |
| Spleen. . . . . . . . . . . | 0 | 0 | 0 | 2 |
| Mesenteric lymph. nodes. . | 0 | 0 | 1 | 0 |

Captain E. C. Ernst. I have some X-ray pictures to show of some investigations made by Captain Eugene Opie and myself on the bodies of British casualties. Captain Opie carefully removed the thoracic contents, exclusive of the heart, and also the mesentery. These were placed on oiled paper on an X-ray plate and a soft picture was taken. The results were most interesting. 70 o/o of the British soldiers revealed calcified tubercle deposits in the nodes of the mesentery. Many of these could only be found later by most careful search. In about 15 o/o deposits were found as shown by these negatives, and later confirmed by dissection in the lungs or tracheo-bronchial lymph nodes as well as in the mesentery. Very few lungs and mesenteries were completely negative. About 15 o/o of the lungs or tracheo-bronchial nodes revealed deposits when the mesentery was completely negative. It has been a curious fact that in our recent work in autopsies of American soldiers only 35 o/o have shown such deposits in the mesentery, and only 15 o/o have shown deposits in the lungs and not in the mesentery.

Colonel Sir Almroth Wright. Colonel Wright after giving an account of his investigations in wound bacteriology said: I should like to know why the tubercle bacillus should attack the upper lobes, and I hardly believe it is yet explained. I have worked very little in tubercle in recent years, and I doubt whether the tubercle bacillus will grow in blood. It is certain that it grows in connective tissues, but these tissues must have been altered before the tubercle bacillus will grow in them. When it comes to a man who has been fighting tubercle in his blood or tissues or any other microbe, you are dealing with a man seriously ill, who is no longer able to put up a good fight. Should you inoculate him with his own microbes you give him what he has been fighting against for years, and I doubt if you would obtain good results by inoculating with this specific microbe. I should feel disposed to give up all inoculation but for the errors I have made in giving a vaccine of the wrong microbe to many patients. I can show you that with any microbe inoculated you get protection against the whole lot; if I inoculate a man against typhoid, I immunize him against streptococcus and staphylococcus. When you are dealing with the tubercle problem, should tuberculin do no good see if you can do any good by inoculating vaccines of other microbes.

11. **How many cases of pulmonary tuberculosis may we expect in the American Expeditionary Forces?**

Lieut.-Colonel E. H. Bruns. At the beginning of the war the Surgeon General saw the necessity of eliminating tuberculosis from the army, and at the same time of preventing soldiers from being discharged for tuberculosis, who did not have this disease. We soon found that little importance could be given to a soldier's history. In examinations we had to depend almost entirely upon physical signs, and on account of the necessity of examining large numbers of men, the examinations had to be rapid. I think the fact that we have very little tuberculosis in the army shows that we eliminated most of it in the beginning. I understand that the first division of regulars and the Rainbow Division, which came to France before being examined for tuberculosis, have had a fair number of cases develop. Medical officers in the United States have not assumed a critical attitude towards the diagnoses made in France, because similar mistakes were naturally made in our camp examinations. It was thought at first that every soldier coming to France would be in danger of contracting tuberculosis from the large number of consumptives reported as being present in the French Army and in the civil population. It was thought, however, that this was simply enemy propaganda, and we were warned against it.

In the first million men examined, about 14,000 were discarded for the same disease by special boards of examiners. In the second draft, the tuberculosis examiner rejected only 6 or 7 per thousand, which is a very small percent., and this was due to the great improvement in the preliminary draft examination. I cannot but think that all these careful examinations have much to do with the small amount of tuberculosis found in our army in France to-day.

Lieut.-Colonel Webb. There are probably many factors to be taken into consideration in regard to the number of cases of pulmonary tuberculosis which we may expect in the American Expeditionary Forces. The incidence and death-rate of young manhood at twenty-five years — the average age of our soldiers — is very much smaller than it used to be. The death-rate at twenty-five per million at that age in the civil population would be about eighteen hundred, and the incidence, therefore, about seven thousand. Had we selected our army from men nearer forty-five years of age, we might have had to allow for double this number. If approximately twenty-one thousand per million were discarded in the United States, it would certainly seem that too many tuberculous soldiers had been discovered. When we recall, too, that we are at present sending home about 1200 per million, per year, we see at once that many cases which were going to break out in France were not discovered, in spite of the large numbers discarded. Of these men, about twice as many are coming from the Service of Supply as from the front. A large percent. of these men are incurable and will probably fall among those who constitute the death-rate at the age of 25. In examining the British death-rates from tuberculosis in England, Brownlee was struck with the fact that whereas in 1861 four thousand per million died of pulmonary tuberculosis at the age of 25, to-day only 1800 die at that age. There has been no such proportional decrease at other age periods. By careful dissection of the figures from separate districts and counties, which make up the general tuberculosis death rate, Brownlee believes that we may be dealing with three types of the human tubercle bacillus.

1. The young adult type, causing death at 25, and constituting almost the only deaths in the Shetland Islands and Ireland.

2. The middle-aged type, causing death at 45, and constituting almost the only deaths in London, Lancashire and some of the central counties.

3. The old-age type, causing death at about 65 years of age. This type would seem to be especially prevalent in Cornwall.

Such an analysis of figures is extremely interesting, but naturally any conclusions will need laboratory confirmation. Such curves have not yet been worked out for the United States, but the curve as a whole for the United States, for which I am indebted to Dr. Farrand of the Rockefeller Commission, is exactly similar to that of Great Britain. We have a different story to tell, however, when it comes to the Negroes. We have nearly five times the mortality at the young-adult period, slightly less mortality at the middle-age period, and one and a half times the mortality at the old-age period. We have therefore had, as would be expected, a little more tuberculosis among the Negroes in France, and although they form a very small percentage of the American Expeditionary Forces, they constitute 14 o/o of the cases of pulmonary tuberculosis returned to the States. Correlating all our postmortem evidence, and all the above facts, we can simply say that the outbreak of pulmonary tuberculosis will be very slight.

As Colonel Bushnell so well points out, in the last three years the tuberculous soldiers found in the French Army did not infect others. We must remember, however, that a large number of our men have come from farms, and just as they have succumbed to epidemics of measles which they had not encountered before, so contact with the tubercle bacillus may directly affect them, as the following figures I have received from Captain Somerville suggest.

Soldiers of the American Expeditionary Forces returned to the United States for Active Pulmonary Tuberculosis.

| | | | |
|---|---|---|---|
| Farmers | 21 o/o | School Teachers | 3 o/o |
| Mechanics | 18 o/o | Chauffeurs | 3 o/o |
| Clerks | 17 o/o | Millhands | 2.5 o/o |
| Laborers | 13 o/o | Miners (Iron and anthracite coal.) | 2 o/o |
| Railroad Employes | 5.5 o/o | | |

It will be noted from the following list that the agricultural states possibly show a greater proportion of cases than the manufacturing states with large cities, like New York, Pennsylvania and Illinois.

| | Average Age. | Number. | | Average Age. | Number. |
|---|---|---|---|---|---|
| Alabama. . . . . | 24 | 30 | Massachusetts. . . | 26 | 15 |
| Arkansas. . . . . | 28 | 6 | Maryland. . . . . | 24 | 8 |
| Colorado. . . . . | 24 | 6 | Minnesota.. . . . | 24 | 12 |
| S. Carolina. . . . | 24 | 12 | Maine. . . . . . | 25 | 3 |
| N. Carolina. . . . | 23 | 8 | New York . . . . | 26 | 58 |
| California. . . . . | 24 | 6 | New Jersey. . . . | 24 | 8 |
| Conn. . . . . . . | 23 | 10 | Nebraska. . . . . | 26 | 6 |
| Delaware. . . . . | 27 | 1 | New Hampshire. . | 28 | 1 |
| S. Dakota . . . . | 24 | 3 | Ohio. . . . . . . | 25 | 18 |
| Florida. . . . . . | 25 | 4 | Oregon. . . . . . | 24 | 4 |
| Georgia. . . . . . | 25 | 17 | Oklahoma . . . . | 25 | 4 |
| Iowa. . . . . . . | 23 | 11 | Pennsylvania . . . | 24 | 33 |
| Idaho.. . . . . . | 19 | 1 | Rhode Island. . . | 24 | 5 |
| Illinois . . . . . . | 25 | 28 | Texas.. . . . . . | 25 | 35 |
| Kentucky. . . . . | 25 | 18 | Tennessee.. . . . | 26 | 12 |
| Kansas. . . . . . | 24 | 4 | Virginia . . . . . | 24 | 16 |
| Louisiana. . . . . | 25 | 8 | Wisconsin . . . . | 21 | 16 |
| Indiana. . . . . . | 23 | 20 | Vermont. . . . . | 29 | 7 |
| Montana. . . . . | 26 | 16 | Wyoming. . . . . | 34 | 1 |
| Michigan. . . . . | 27 | 12 | West Virginia. . . | 26 | 10 |
| Mississippi . . . . | 27 | 14 | | | |

It is impossible naturally to state what proportion this constitutes of the troops from each state.

Apart from these figures outdoor workers constitute 50.4 o/o and indoor workers constitute 49.6 o/o of those returned for pulmonary tuberculosis.

12. **Can these cases of pulmonary tuberculosis be detected earlier than has been done up to the present time?**

MAJOR RIST. My experience in the French Army has been that a great number of people who have been rejected from the army previous to the war on account of tuberculosis are doing well now, although they have never been treated in a sanatorium. They have been working hard in the fields and factories, and possibly, therefore, were not tuberculous. It will certainly be interesting to follow up the cases in the United States which have been rejected, and to find out what percentage have really ultimately become tuberculous. In the French Army, soldiers who were discovered to be really tuberculous were generally advanced cases — severe cases from the outset. Among the sick soldiers who come to the tuberculosis centers in France, the larger portion come with an incorrect diagnosis. But in those who are really tuberculous we very seldom find men showing classical signs of incipient tuberculosis. Generally there is the involvement of one whole lobe at least, and this agrees with the observation of Colonel Webb in the American Army. To me it seems to indicate that what we have considered as incipient tuberculosis is tuberculosis in a more or less advanced stage. I do not think cases of actual pulmonary tuberculosis can be detected much earlier than has been done; they might be detected three or four weeks earlier, but certainly not one year earlier.

Lieut.-Colonel Webb. It has been the complaint of all armies engaged in this war that the men with pulmonary tuberculosis were in an advanced stage when detected. The men arriving at our centers of observation either have a great deal of pulmonary tuberculosis, as stated above, or else none at all, such as bronchitis and pneumonia convalescence.

A somewhat increasing incidence of pulmonary tuberculosis, appearing in some negro labor battalions stationed at Bordeaux, led us recently to examine 3500 of these men. Major Bjerving reports that only one case was found with sputum positive to tubercle, but that 22 cases were discarded for observation. Stewart of Canada recently expressed the opinion at the American National Tuberculosis Association that 25 years ago the diagnosis of tuberculosis was made conservatively, five years ago made freely. Now again it is being made conservatively, and we are really passing through an age of scepticism.

It is easy to see from the reports of Colonel Bushnell's analysis given above that the peri-bronchial or closed type of pulmonary tuberculosis can easily escape detection, and, if we accept this as being incipient, we must conclude that without a series of X-ray examinations incipient tubercle will escape detection.

I trust the Surgeon-General will attempt a series of X-ray and clinical examinations of some ten thousand soldiers, and that these examinations may be repeated every two weeks. I believe from such a study changes might be followed in the lungs of those who will eventually prove tuberculous, and from the study important answers to this last question will be derived.

(*The report of the Conference on Surgery in Battle Areas has been lost in transportation. It is hoped that it may be found in time for the February number.*)

